# Design a Friendly Nanoscale Chemical Sensor Based on Gold Nanoclusters for Detecting Thiocyanate Ions in Food Industry Applications

**DOI:** 10.3390/bios14050223

**Published:** 2024-04-30

**Authors:** Reham Ali, Sayed M. Saleh

**Affiliations:** 1 Department of Chemistry, College of Science, Qassim University, Buraidah 51452, Saudi Arabia; e.saleh@qu.edu.sa; 2 Chemistry Department, Faculty of Science, Suez University, Suez 43518, Egypt; 3 Department of Petroleum Refining and Petrochemical Engineering, Faculty of Petroleum and Mining Engineering, Suez University, Suez 43721, Egypt

**Keywords:** gold nanoclusters, thiocyanate detection, fluorescent nanosensor, nanofabrication, food and nutrition, environment and human health

## Abstract

The surfactant cetyltrimethylammonium bromide (CTAB) induces the aggregation of gold nanoclusters (GNCs), leading to the development of a proposed fluorometric technique for detecting thiocyanate (SCN^−^) ions based on an anti-aggregation mechanism. This approach is straightforward to execute, highly sensitive, and selective. A significant quenching effect occurs in fluorescence upon using the aggregation agent CTAB in GNCs synthesis, resulting in a transition from intense red fluorescence to dim red. The decrease in fluorescence intensity of GNCs in the presence of CTAB is caused by the mechanism of fluorescence quenching mediated by aggregation. As the levels of SCN^−^ rise, the fluorescence of CTAB-GNCs increases; this may be detected using spectrofluorometry or by visually inspecting under UV irradiation. The recovery of red fluorescence of CTAB-GNCs in the presence of SCN^−^ enables the precise and discerning identification of SCN^−^ within the concentration range of 2.86–140 nM. The minimum detectable concentration of the SCN^−^ ions was 1 nM. The selectivity of CTAB-GNCs towards SCN^−^ ions was investigated compared to other ions, and it was demonstrated that CTAB-GNCs exhibit exceptional selectivity. Furthermore, we believe that CTAB-GNCs have novel possibilities as favorable sensor candidates for various industrial applications. Our detection technique was validated by analyzing SCN^−^ ions in milk samples, which yielded promising results.

## 1. Introduction

The thiocyanate ion (SCN^−^) is an important biomarker in the food industry to provide the safety of consumers. SCN^−^ is used as a preservative material that is added to dairy products to prolong their shelf-life; it is an anti-bacterial agent. When the SCN^−^ concentration in these highly nutritional products increases, it poses high health risks. The identification of SCN^−^ is of utmost importance. It has been found that the natural concentration of SCN^−^ in milk is around 40–67 μM, which is equivalent to 5–8.5 mg L^−1^ [1]. Abuse of SCN^−^ potentiates the incurrence of developing hypothyroidism. It disrupts thyroid hormone synthesis by interfering with iodide uptake in the human body, resulting in goiters. Diminished amounts of iodide and decreased transport of iodide into the thyroid follicular cell will reduce the amount of thyroxine produced by the thyroid gland. High concentrations of thiocyanate also cause cardiac and neurologic toxicity, vertigo, nasal bleeding, unconsciousness, and even unconsciousness in extreme cases [2,3]. Notwithstanding, SCN^−^ ions have an essential role in the biosynthesis of hypothiocyanite by a lactoperoxidase enzyme [4]. Thus, SCN^−^ deficiency in the human body damages the host defense system [5,6]. Detection of the concentrations of SCN^−^ ion has become of serious importance, and there is a need for a rapid and sensitive analytical protocol that can be applied to the health and safety of communities worldwide.

Several different methods of detection, including voltammetry [7], high-performance liquid chromatography (HPLC) [8], ion chromatography [9,10], capillary zone electrophoresis [11], flow injection analysis [12], colorimetry [13,14], and fluorescence approaches [15,16], are utilized. The detection of SCN^−^ has been accomplished by using surface-enhanced Raman scattering (SERS) [17,18]. Although these techniques are extremely helpful for detecting SCN^−^, some of them need the use of complex instruments, processes that take a significant amount of time, the presence of trained personnel, and even chemicals that are either expensive or hazardous for the pretreatment of the sample [19]. Therefore, developing a SCN^−^ sensor that has the features of simplicity, reliability, and sensitivity is urgently needed for an environmentally friendly method. Optical sensors have garnered greater attention due to their numerous benefits, including the ability to conduct on-site analysis and visualization; their simplicity and sensitivity; and the absence of skilled specialists [20,21,22,23,24].

Florescent nanosensor-based analytical detection has a wide application in different fields. In particular, gold nanoclusters (GNCs) have emerged as a very attractive possibility for use in chemical, medicinal, and biological applications. Due to their distinct advantages over other nanomaterials and conventional organic dyes, they have found extensive use as a nanoprobe for chemical sensing of different analytes [25,26,27,28,29,30]. Ultra-small GNCs, approximately 3 nm in size, have molecular-like characteristics referring to the extreme quantum boundary influence that initiates the continuous energy bands to split into discrete energy levels. Therefore, AuNCs possess common features, such as the HOMO-LUMO transition, photoluminescence (PL), lacking an SPR peak, electromagnetism, redox behavior, and molecular chirality [31,32]. Their characteristics include a high level of photoluminescence, a significant Stokes shift, chemical stability, easy synthesis, and non-toxicity. The fluorescence signals of these minuscule particles may be adjusted to different wavelengths within the visible to near-infrared range, depending on the nanocluster’s size and the atom number they contain [33,34]. The size depends on the reducing and protecting agent employed in the reduction process, which aids in achieving colloidal stability and inhibiting aggregation. Most ligand-stabilized gold nanoclusters have been utilized for sensing purposes and applications such as detecting certain metal ions or molecules. This is achieved by the targeted quenching of the intense fluorescence emitted by GNCs in the presence of these ions or molecules [35,36]. Aggregation of GNCs causes a change in the emission of the colloidal solution, decreasing the fluorescence. The size of the GNCs determines the emission change due to changes in the inter-particle distance between the atoms. Several optical sensors have been created based on their anti-aggregation to enhance selectivity and minimize false-positive signals [37,38]. This contrasts with the numerous sensors based on nanoparticle aggregation [39,40]. In systems designed to prevent the aggregation of GNCs, introducing an aggregation agent first causes the GNCs to aggregate by altering the spacing between their particles. However, because the target analyte has a great binding affinity to the aggregating agent, it will inhibit the gold nanoparticles’ aggregation. Recent investigations have shown that cetyltrimethylammonium bromide (CTAB), a cationic surfactant, possesses advantageous characteristics, such as a positive charge that can assemble and synthesize different types of nanoparticles [41,42].

**Scheme 1:**
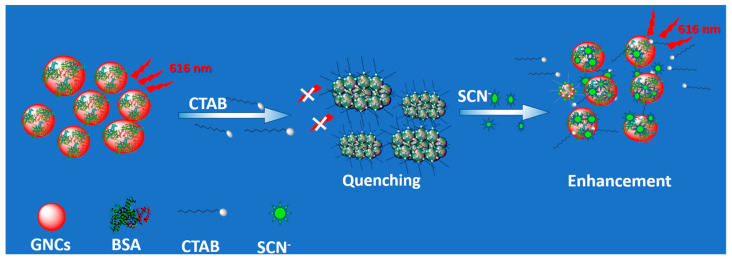
The CTAB-GNCs’ synthesis and their application for sensing SCN^−^.

In this article, a colorimetric technique that is both simple and extremely sensitive is suggested for the detection of SCN^−^. This strategy is based on the SCN^−^-prevented aggregation of CTAB-GNCs. A decrease in the fluorescence intensity due to the incorporation of CTAB into GNCs caused the aggregation of GNCs. In the meantime, the aggregation of CTAB-GNCs was halted in the presence of SCN^−^ due to the competitive reaction between SCN^−^ and the aggregation agent CTAB, as depicted in Scheme 1. By increasing the SCN^−^ concentration, the naked eye can easily notice the red fluorescence turn-on under ultraviolet irradiation without using expensive and complex apparatuses.

## 2. Materials and Methods

### 2.1. Reagents and Materials

The surfactant cetyltrimethylammonium bromide (CTAB), Tetrachloroauric(III) acid trihydrate (HAuCl_4_·3H_2_O, 99%), and sodium thiocyanate were obtained from (Sigma-Aldrich, St. Louis, MO, USA). The Millipore (Milli-Q system, Burlington, MA, USA) was used to provide deionized (DI) water for all experiments. All the other chemicals were of analytical reagent grade and used as received without further purification. All aqueous stock solutions of analyte were prepared in water.

### 2.2. Instruments

A UV-visible spectrophotometer from the Evolution^TM^ 200 series (Thermo Fisher Scientific, Madison, WI, USA) was used to measure the absorption spectra with a quartz cell that was 1 cm in diameter. Using a quartz luminescence-free cell with a 1 cm path length and 5 nm band pass excitation and emission filters, the fluorescence spectra of BSA-GNCs were measured with a JASCO FP6300 spectrofluorometric connected to an effective temperature controller. (JEOL-100S, Tokyo, Japan), recorded high-resolution transmission electron microscopy (HRTEM) images of BSA-GNCs at 200 kV. Before HRTEM, the BSA-GNCs solutions were sonicated, dripped onto copper grids covered with carbon, and dried at ambient temperature. The Zeta sizer Nano ZS90 gear (Malvern Panalytical Ltd., Malvern, UK) was used to capture dynamic light scattering (DLS). A Perkin Elmer FT-IR spectrometer was used to measure the Fourier-transform infrared spectra. Utilizing monochromatic X-ray Al K-alpha radiation with a spot size of 30 µm and a pressure of 10^−9^ mbar, XPS was conducted on a K-ALPHA (Thermo Fisher Scientific, Waltham, MA, USA) with a full-spectrum pass energy of 200 eV and a narrow-spectrum energy of 50 eV.

### 2.3. Synthesis of CTAB-Gold Nanoclusters (CTAB-GNCs)

The CTAB-GNCs were synthesized using the reported method with some modifications [43]. The glassware used to synthesize GNCs was cleansed with Aqua Regia, rinsed with ethanol and ultrapure water, and left to dry in the open air. Under vigorous stirring, 5 mL of a 10 mM aqueous solution of HAuCl_4_ at 37 °C was added to a 5 mL aqueous solution of BSA at a concentration of 50 mg/mL. Subsequently, a 0.5 mL, 1 M NaOH solution was introduced to the preceding solution after a duration of 2 min, using the most current procedure [30]. The reaction was conducted with vigorous agitation at a temperature of 37 °C for a duration of 4 h. A total of 1 mL of CTAB (5 mg/mL) was added to the mixture, and the reaction lasted 20 h. The color transitioned from a light yellow to bale brown during this time. The produced CTAB-GNCs underwent dialysis in ultra-high-purity water, which was changed every 8 h for approximately 24 h. This process was carried out to eliminate all small-molecular contaminants and obtain the purified product. The solution of CTAB-GNCs obtained after dialysis was kept at a temperature of 4 °C for future use. The concentration of the generated CTAB-GNCs was 4.3 mM, assuming a complete reduction of all Au(III) ions during the nanocluster synthesis.

### 2.4. Synthesis of Gold Nanoclusters (GNCs)

For the preparation of GNCs, the same procedure was carried out but without adding CTAB, and the reaction proceeded to 24 h to compare their fluorescence.

### 2.5. Detection of SCN^−^ Ions Using CTAB-GNCs

The prepared CTAB-GNCs were used for sensing SCN^−^ ions. Using 100 µL of CTAB-GNCs in a phosphate buffer solution (pH 7.5, 10 mM), various volumes of 0.5 mM of SCN^−^ were incubated for 1 min with the solutions. The solutions were examined using fluorescence spectroscopy (λ_exc_/λ_em_ = 465/613 nm). Other species were tested with the same procedure, including cations, anions, and molecules, to study the selectivity and sensitivity of CTAB-GNCs to SCN^−^.

### 2.6. Real Sample Preparation

The standard protocol [44] treated liquid milk samples to eliminate the proteins. Two grams of milk product was added to 10.0 mL of acetonitrile and 3 mL of 30% trichloroacetic acid. The mixture was sonicated for 10 min and centrifuged at 6000 rpm for 20 min. The pH of the filtrate was adjusted to 7.5 using phosphate buffer. A total of 2 mL of the solution was mixed with 100 µL of CTAB-GNCs and spiked with SCN^−^ at concentrations of 20, 60, 100, and 140 nM.

## 3. Results

### 3.1. Synthesis and Optical Properties of CTAB-GNCs

**Figure 1:**
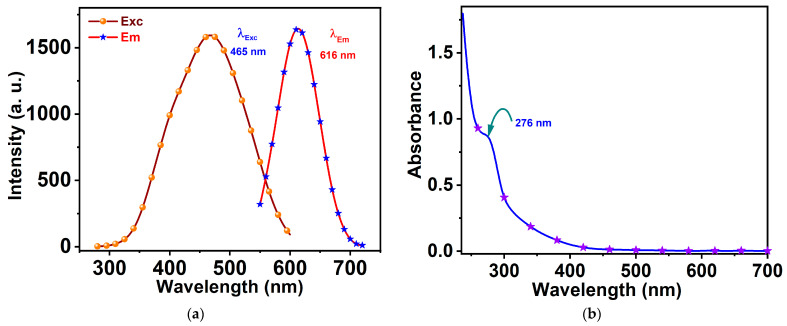
(**a**) Fluorescence emission of as-prepared GNCs at 616 nm under excitation at 465 nm. (**b**) Absorption spectra of aqueous solutions of GNCs.

BSA is widely used for the synthesis of GNCs. It plays a crucial role in stabilizing GNCs and enhancing their fluorescence. The molecular chain of BSA in the presence of NaOH was used as a reducing and stabilizing agent for Au(III) ions, which were reduced to Au(0) by the tyrosine residue of BSA as the nanoclusters formed. The sensitized GNCs exhibit strong and bright-red fluorescence. They have been serving as chemical sensors in many fields and have different applications, even based on quenching approaches or enhancements of fluorescence in the presence of the analyte. The unique features include aqueous solubility, ultra-small size, high quantum yield with a large Stokes shift (150 nm), excellent photostability, and biocompatibility, prioritizing them to be a superior sensing material for SCN^−^ detection. The synthetic GNCs showed absorbing characteristics and light-emitting at 276 and 616 nm (under excitation with 465 nm), respectively, as shown in Figure 1a,b after 24 h at 37 °C. The absence of surface plasmon resonance absorption by the BSA-GNCs solution suggests no gold nanoparticles were formed. Absorption at 276 nm, on the other hand, suggested that it had features characteristic of molecules.

**Figure 2:**
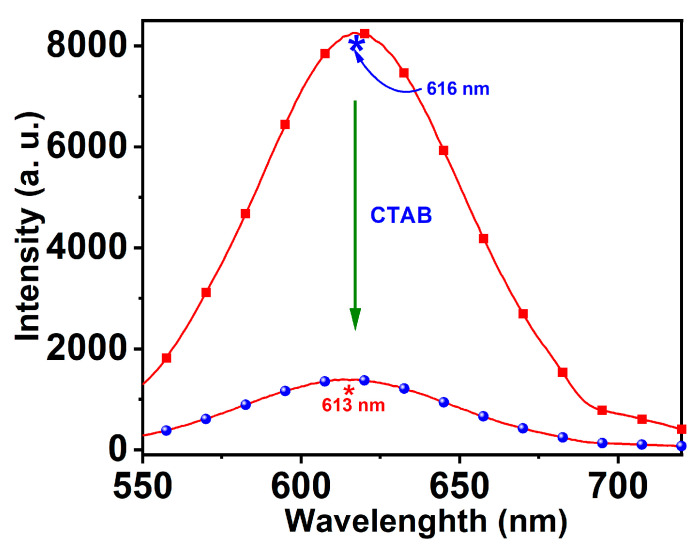
Fluorescence of GNCs and CTAB-GNCs under excitation at 465 nm.

CTAB is a cationic surfactant with a positively charged hydrophilic head and neutral hydrophobic tail. In the synthesis process, in the presence of CTAB, it can absorb the surface of GNCs to form a bilayer. It can readily bind to GNCs, causing aggregation. The aggregation effect of different cationic surfactants on the growth of GNCs was previously reported [45,46]. As the CTAB concentration increases, aggregation increases due to the increase in the GNC’s aggregation and size. In our work, CTAB was added to the GNCs during the synthesis process to ensure its adsorption on the direct growth of the GNCs. By comparing the fluorescence of CTAB-GNCs and GNCs prepared by the same procedure but without adding CTAB, quenching in fluorescence occurred due to aggregation-induced fluorescence quenching of GNCs with a very small blue shift (3 nm) (see Figure 2).

### 3.2. Studying the Optimal Concentration of CTAB

**Figure 3:**
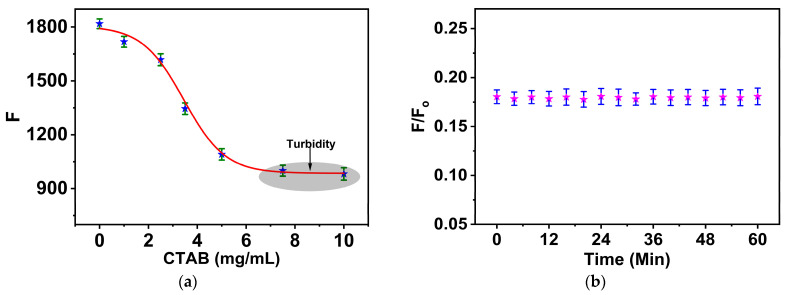
(**a**) The effect of CTAB concentration on the emission of GNCs. (**b**) Recognition of the F/F_o_ of CTAB-GNCs over time.

Different concentrations of CTAB were used as an aggregating agent in the synthesis of CTAB-GNCs to attain the optimal concentration. In total, 1, 2, 5, 7.5, and 10 mg/mL of CTAB were studied, where increasing the amount of CTAB to more than 5 mg/mL caused turbidity that increased with the increase in the CTAB concentrations and very low fluorescence to be obtained. Meanwhile, concentrations under 5 mg/mL had a weak significant quenching effect on GNCs fluorescence. As a result, 5 mg/mL was chosen as the optimal concentration of CTAB; see Figure 3a.

To study the stability of CTAB-GNCs’ fluorescence signal in solution with time, the fluorescence intensity of the CTAB-GNCs was measured at different intervals. Fluorescence is indicated by the letters F_o_ at 0 times, while it is indicated as F after different intervals. We have measured the F/F_o_ at various time intervals. Figure 3b shows that the fluorescence of CTAB-GNCs is stable over time. Additionally, a steady level of fluorescence was seen in the produced GNCs solution even after six months of dark storage, suggesting that the solution is stable over the long term. These probes are designed to be water-soluble fluorescent GNCs. Therefore, this approach would be an excellent choice for quick SCN^−^ evaluation.

### 3.3. GNCS and CTAB-GNCs Characterization

Due to their unique size-dependent properties, investigating the aggregation of gold nanoclusters (GNCs) is an intriguing field of study with substantial ramifications across diverse scientific and technological domains. The potential applications of GNCs based on aggregation-caused quenching in biosensing, bioimaging, drug delivery, and therapy have been intensively studied. Aggregation-caused quenching is a fascinating phenomenon linked to GNCs, and it pertains to transforming molecules that are intensely luminescent states into non-emissive or weakly emissive when aggregating [47,48].

**Figure 4:**
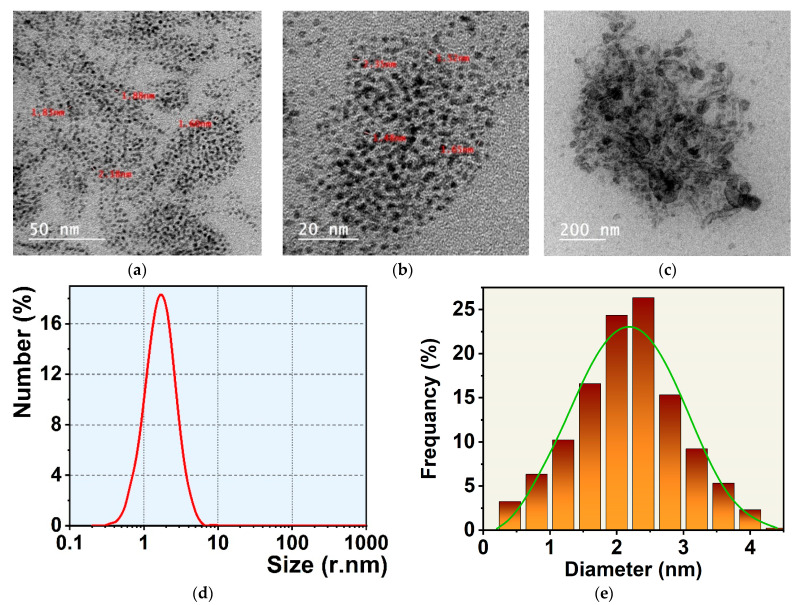
(**a**,**b**) HRTEM images of the GNCs; (**c**) HRTEM images of the CTAB-GNCs; (**d**,**e**) DLS measurements and size distribution histograms of the GNCs.

The BSA-stabilized nanoclusters GNCS and CTAB-GNCs were characterized using HRTEM to ensure that nanoclusters were successfully synthesized. The size of BSA-GNCs was recorded to be 1.4 nm, confirmed by the DLS and fluorescence measurements (see Figure 4), and no aggregation was observed. However, in the case of CTAB-GNCs, the transmission electron microscopy data demonstrated the aggregation process of GNCs in the presence of CTAB, which revealed that incorporating CTAB molecules results in the formation of larger clusters and the aggregation of GNCs (see Figure 4). This indicates the binding of CTAB to BSA, which induced aggregation of GNCs.

**Figure 5:**
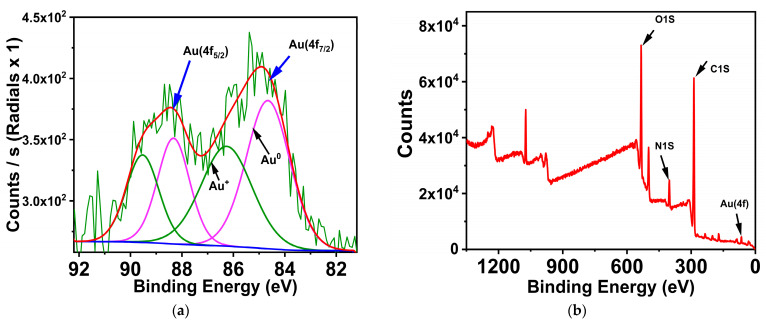
(**a**) XPS spectra of Au 4f for CTAB-GNCs; (**b**) The whole XPS spectrum of CTAB-GNCs.

X-ray photoelectron spectroscopy (XPS) was employed to study the overall electronic structure of the CTAB-GNCs, including the surface and the oxidation state of Au (4f). Figure 5a shows two peaks centered at 83.9 eV and 87.5 eV corresponding to the electronic states of Au 4f_7/2_ and Au 4f_5/2_, respectively. The Au 4f_7/2_ spectrum (red curve) is deconvoluted into two peaks at 84.0 eV and 86.0 eV (purple and green curves), corresponding to the reduction of Au(III) into Au(0) and Au(I), respectively. However, Au(I) content was found to be less than Au (0) content [49]. Moreover, the Au 4f_5/2_ peak is deconvoluted into two peaks at 88.5 eV and 89.6 eV, corresponding to the existence of Au(0) and Au(I), respectively. The photoemission spectra of C(1s), N(1s), and O(1s) characterized that CTAB is bound to BSA-GNCs, as shown in Figure 5b.

### 3.4. SCN^−^ Detection Mechanism Based on CTAB-GNCs

The aggregation of GNCs locks the luminescent properties due to their unique size-dependent properties, which renders them extraordinarily versatile for an extensive array of applications. The managed aggregation of GNCs is a cutting-edge technique in nanotechnology that holds great potential for future innovations, including developing sophisticated illumination systems and preventing cancer [50]. The capacity to control how they aggregate presents novel opportunities for developing materials with tailored optical and electronic characteristics, facilitating significant advancements in fundamental research and pragmatic implementations. Anti-aggregation-induced emission, a fascinating phenomenon linked to GNCs, pertains to transforming molecules that are otherwise non-emissive or weakly emissive into intensely luminescent states when aggregating eliminates by adding analyte. In brief, the luminescent properties of these nanoclusters are unlocked by the anti-aggregation of GNCs, which renders them extraordinarily versatile for an extensive array of applications [50].

CTAB-GNCs were chosen as promising candidates for use as nanoprobes to detect the SCN anion. This anion is known to have a high affinity for coordinating with Au atoms on the surface of GNCs, resulting in the departure of CTAB from the surface of GNCs. Eventually, CTAB tends to leave the GNCs’ surface and allow the nanoclusters to be dispersed again in the solution associated with restoring their fluorescence. Consequently, the fluorescence intensity of the GNCs increases; thus, the detection of SCN^−^ could be realized. The extreme advantages of the fluorescence turn-on sensing mechanism concerning the turn-off mechanism are the low detection limit and the dark fluorescence background of the chemosensor [51]. These factors decrease the probability of a pseudo signal and increase both sensitivity and selectivity. Moreover, the visual sensing-based naked eyes would provide a strong tool with high sensitivity and selectivity towards the specified analyte.

**Figure 6:**
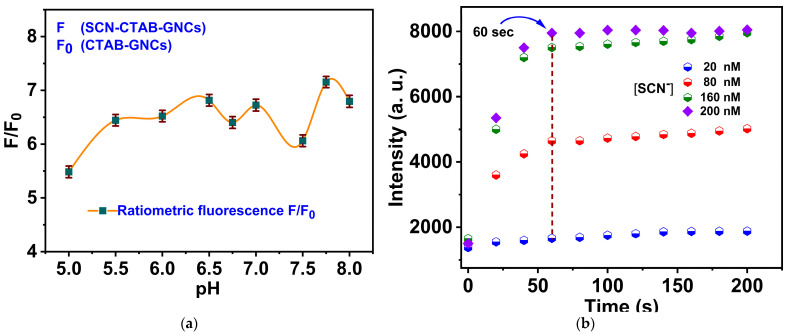
(**a**) The ratiometric intensities of CTAB-GNCs in the absence (synthesis process) and in the presence of SCN^−^ (sensing process) versus the pH of the medium; (**b**) the relation between the fluorescence intensity versus time.

Different experiments were carried out to study the optimal conditions of SCN^−^ sensing. The CTAB-GNCs solution was studied at various pH levels in SCN-presence. The fluorescence of the CTAB-GNCs solution with the addition of 160 nM of SCN^−^ and at different pH levels (adjusted by 10 mM phosphate buffer) is shown in Figure 6a. At pH 7.5, the fluorescence of GNCs solutions reached the maximum value. Therefore, 7.5 was chosen as the optimal pH for the other investigations. The fluorescence spectra of the CTAB-GNCs were further optimized with time. Figure 6b shows the time-dependent changes in the fluorescence of CTAB-GNCs solutions in the absence and presence of SCN^−^. The anti-aggregation of CTAB-GNCs is almost 90% completed within 60 s. Therefore, all the fluorescence measurements were performed after 60 s.

**Figure 7:**
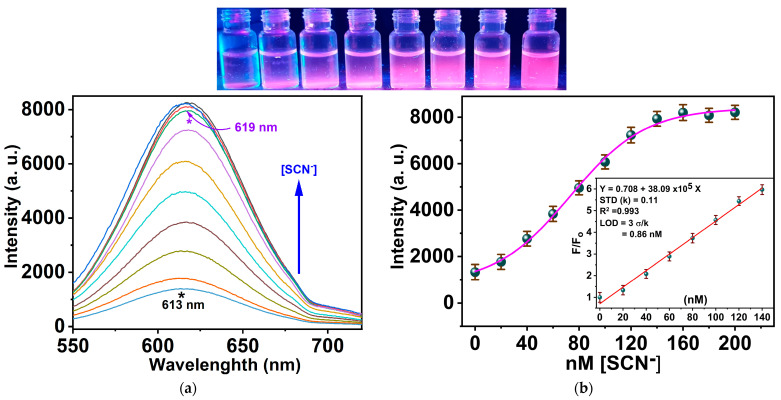
(**a**) Fluorescence intensity of CTAB-GNCs in the presence of different concentrations of SCN^−^ (0–200 nM). (**b**) Relation between the fluorescence of CTAB-GNCs versus SCN^−^ concentrations.

Upon the titrimetric fluorescence study of SCN^−^ in the range of 0–200 nM to an aqueous solution of CTAB-GNCs in an adjusted phosphate buffer (10 mM, pH 7.5), it has been found that the fluorescence of CTAB-GNCs at 613 nm increased with the increase in the concentration of SCN^−^, as shown in Figure 7a. By further addition of SCN^−^, a continuous increase is observed in the fluorescence until no signal alterations on the gradual addition of SCN^−^ at 200 nM. The enhancement of GNCs can be attributed to the anti-aggregation mechanism. Figure 7b illustrates the relationship between the fluorescence intensity of CTAB-GNCs at 613 nm and the various SCN concentrations in a 0–200 nM concentration range. The nanosensor limit of detection (LOD) was established to be 0.86 nM.

In analytical chemistry, the Limit of Detection (LOD) is an essential parameter denoting the minimum concentration of a substance that can be detected with dependability through an analytical procedure. The LOD is computed utilizing the equation LOD = 3σ/k, where σ represents the response’s standard deviation, and k denotes the slope of the calibration curve [52]. y = a + b*x, k = 0.11, δ = 38.09x 10^5^, R^2^ = 0.993. The LOQ was calculated to be 2.86 nM. The formula utilized in this context is based on the notion that the LOD, or critical value, signifies when a quantified value diverges statistically from the empty measurement with a 95% confidence level. A factor of 3 guarantees that the LOD is adequately elevated above the noise level to enable dependable detection [53,54]. Particularly in fields where detecting low concentrations of analytes is frequently critical, such as food safety testing, clinical diagnostics, and environmental monitoring, this calculation is indispensable for ensuring the precision and dependability of analytical measurements.

### 3.5. Interference and Selectivity of CTAB-GNCs toward SCN^−^

**Figure 8:**
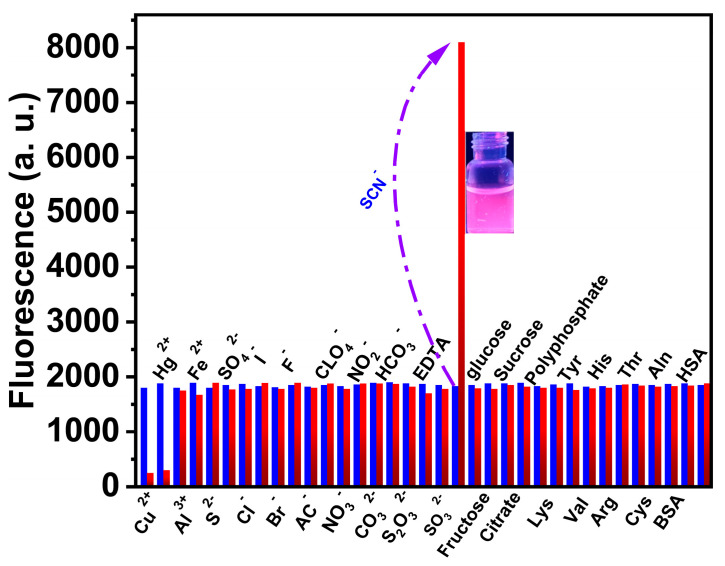
The fluorescence intensities of the GNCs alone (blue bar) and their intensities in the presence of SCN^−^, different cations, anions, and molecules (red bar).

The selectivity of detecting SCN^−^ based on the fluorescence enhancement effect of CTAB-GNCs has been studied in the presence of the main relevant anions and cations. It was found that Cu^2+^ and Hg^2+^ ions have a significant quenching effect on the fluorescence of CTAB-GNCs, which is related to metallophilic interaction between Hg^2+^–Au^+^ in the existence of Hg^2+^ and intermolecular charge transfer in the case of Cu^2+^ [55]. However, no effect in the fluorescence was observed in the presence of other species, as shown in Figure 8, and only SCN^−^ caused a significant enhancement in the fluorescence of CTAB-GNCs. We reason that as SCN^−^ has a much higher negative charge density than other species, it has a stronger affinity towards Au atoms and Au(I) ions on GNCs’ surface and induces the anti-aggregation effect of the CTAB-GNCs. All these results illustrate the presented method for sensing SCN^−^, which exhibits excellent selectivity and can be applied in many applications.

### 3.6. Applications

This study aims to develop an optical chemosensor capable of detecting harmful SCN^−^ ions efficiently to preserve the quality of food and the general population’s health. Milk samples were used to evaluate the availability of the assay and its application to confirm the usefulness of our technology in determining whether or not it is possible to identify SCN^−^ levels in milk samples. After removing proteins from milk samples using the pretreatment method, the samples were combined with CTAB-GNCs to facilitate the fluorescence measurements. The milk samples were measured first without adding SCN^−^ solutions; however, no fluorescence change was obtained, indicating the absence of SCN^−^ in the milk samples. The same experiment was repeated after the samples were spiked with several SCN^−^ concentrations (20, 60, 100, and 140 nM). The higher concentration of spiking SCN^−^ was shown to increase the luminescence intensity of the CTAB-GNCs, as evaluated by the experiment. An overview of the findings may be found in Table 1. The results for recovery ranged from 98 to 107%. The findings demonstrate that the suggested detection approach, based on our optical nanosensor (CTAB-GNCs), has a considerable influence on the quantitative measurement of SCN^−^ in practical applications that is reliable and appropriate.

## 4. Conclusions

We present the first SCN^−^ nanosensor based on the red-emitting gold nanoclusters stabilized by BSA. The synthesized GNCs were modulated by CTAB, which quenches the red fluorescence based on a mechanism involving aggregation-induced fluorescence quenching. The resulting nanoclusters have excitation/emission at 465/613 nm. The red-emitting CTAB-GNCs were easily restored again by adding different SCN^−^ concentrations into the solution that inhabits the aggregation caused by CTAB. The optimal conditions for the synthesis of clusters and the optimal condition for the sensing process of SCN^−^ were also studied. The fluorescence “turn-on” enables a reliable sensitive SCN^−^ detection in the 3.33–160 nM range. The synthesized GNCs have been used as fluorescence nanoprobes for low-level detection of SCNs in milk samples.

## Figures and Tables

**Table 1:** Detection of SCN^−^ in milk samples using CTAB-GNCs (n = 3).

Samples	Added SCN^−^ (nM)	Found (nM)	RSD (%)	Recovery (%)
Liquid milk	20.0	21.2	3.6	106
	60.0	64.2	3.1	107
	100.0	103.2	2.2	103
	140	137.3	1.7	98

## Data Availability

Data are contained within the article.

## References

[1] Feng Y., Mo R., Wang L., Zhou C., Hong P., Li C. (2019). Surface enhanced Raman spectroscopy detection of sodium thiocyanate in milk based on the aggregation of Ag nanoparticles. Sensors.

[2] Chi H., Cui X., Lu Y., Yu M., Fei Q., Feng G., Shan H., Huan Y. (2022). Colorimetric determination of cysteine based on Au@ Pt nanoparticles as oxidase mimetics with enhanced selectivity. Microchim. Acta.

[3] Willemin M.E., Lumen A. (2019). Characterization of the modes of action and dose-response relationship for thiocyanate on the thyroid hormone levels in rats using a computational approach. Toxicol. Appl. Pharmacol..

[4] Conner G.E., Wijkstrom-Frei C., Randell S.H., Fernandez V.E., Salathe M. (2007). The lactoperoxidase system links anion transport to host defense in cystic fibrosis. FEBS Lett..

[5] Banfi B. (2007). A novel host defense system of airways is defective in cystic fibrosis: Update. Am. J. Respir. Crit. Care Med..

[6] Xu Y., Szép S., Lu Z. (2009). The antioxidant role of thiocyanate in the pathogenesis of cystic fibrosis and other inflammation-related diseases. Proc. Natl. Acad. Sci. USA.

[7] Li D., Xie F., Zhang J. (2018). Voltammetric behaviors and determination of thiocyanate on multiwalled carbon nanotubes-cetyltrimethylammonium bromide modified electrode. Electroanalysis.

[8] Akiyama H., Matsuoka H., Okuyama T., Higashi K., Toida T., Komatsu H., Sugita-Konishi Y., Kobori S., Kodama Y., Yoshida M. (2015). The acute encephalopathy induced by intake of sugihiratake mushroom in the patients with renal damage might be associated with the intoxication of cyanide and thiocyanate. Food Saf..

[9] Jiang B., Zhong S., Yu H., Chen P., Li B., Li D., Liu C., Feng Z., Tian B. (2021). Aqueous Two-Phase System–Ion Chromatography for Determination of Thiocyanate in Raw Milk. Separations.

[10] Destanoğlu O., Gümüş Yılmaz G. (2016). Determination of cyanide, thiocyanate, cyanate, hexavalent chromium, and metal cyanide complexes in various mixtures by ion chromatography with conductivity detection. J. Liq. Chromatogr. Relat. Technol..

[11] Lobo-Júnior E.O., LS Chagas C., Coltro W.K. (2018). Determination of inorganic cations in biological fluids using a hybrid capillary electrophoresis device coupled with contactless conductivity detection. J. Sep. Sci..

[12] Ohshima T., Kagaya S., Gemmei-Ide M., Cattrall R.W., Kolev S.D. (2014). The use of a polymer inclusion membrane as a sorbent for online preconcentration in the flow injection determination of thiocyanate impurity in ammonium sulfate fertilizer. Talanta.

[13] Song J., Huang P.C., Wan Y.Q., Wu F.Y. (2016). Colorimetric detection of thiocyanate based on anti-aggregation of gold nanoparticles in the presence of cetyltrimethyl ammonium bromide. Sens. Actuators B Chem..

[14] Peng C.F., Pan N., Zhi-Juan Q., Wei X.L., Shao G. (2017). Colorimetric detection of thiocyanate based on inhibiting the catalytic activity of cystine-capped core-shell Au@ Pt nanocatalysts. Talanta.

[15] Sundaram E., Servarayan K.L., Vasantha V.S. (2022). Optical detection of thiocyanate in human saliva based on the colorimetric response of (2-(2-hydroxyphenyl)-1H-benzo [d] imidazol-5-yl)(phenyl) methanone (HBPM)/Co^2+^ ions conjugate. Spectrochim. Acta Part A Mol. Biomol. Spectrosc..

[16] Ahmed S.R., Sherazee M., Srinivasan S., Rajabzadeh A.R. (2022). Positively charged gold quantum dots: An nanozymatic “off-on” sensor for thiocyanate detection. Foods.

[17] Wirojsaengthong S., Aryuwananon D., Aeungmaitrepirom W., Pulpoka B., Tuntulani T. (2021). A colorimetric paper-based optode sensor for highly sensitive and selective determination of thiocyanate in urine sample using cobalt porphyrin derivative. Talanta.

[18] Bai X.R., Zhang L., Ren J.Q., Shen A.G., Hu J.M. (2021). The small silver nanoparticle-assisted homogeneous sensing of thiocyanate ions with an ultra-wide window based on surface-enhanced Raman-extinction spectroscopy. Anal. Methods.

[19] Guo L., Wang Y., Zheng Y., Huang Z., Cheng Y., Ye J., Chu Q., Huang D. (2016). Study on the potential application of salivary inorganic anions in clinical diagnosis by capillary electrophoresis coupled with contactless conductivity detection. J. Chromatogr. B.

[20] Ali R., Alminderej F.M., Saleh S.M. (2020). A simple, quantitative method for spectroscopic detection of metformin using gold nanoclusters. Spectrochim. Acta Part A Mol. Biomol. Spectrosc..

[21] Saleh S.M., Alminderej F.M., Ali R., Abdallah O.I. (2020). Optical sensor film for metribuzin pesticide detection. Spectrochim. Acta Part A Mol. Biomol. Spectrosc..

[22] Saleh S.M., Ali R., Hegazy M.E.F., Alminderej F.M., Mohamed T.A. (2020). The natural compound chrysosplenol-D is a novel, ultrasensitive optical sensor for detection of Cu(II). J. Mol. Liq..

[23] Saleh S.M., Ali R., Alminderej F., Ali I.A. (2019). Ultrasensitive optical chemosensor for Cu(II) detection. Int. J. Anal. Chem..

[24] Saleh S.M., Ali R., Ali I.A. (2017). A novel, highly sensitive, selective, reversible and turn-on chemi-sensor based on Schiff base for rapid detection of Cu (II). Spectrochim. Acta Part A Mol. Biomol. Spectrosc..

[25] Saleh S.M., Almotiri M.K., Ali R. (2022). Green synthesis of highly luminescent gold nanoclusters and their application in sensing Cu(II) and Hg(II). J. Photochem. Photobiol. A Chem..

[26] Zuber G., Weiss E., Chiper M. (2019). Biocompatible gold nanoclusters: Synthetic strategies and biomedical prospects. Nanotechnology.

[27] Ali R., Saleh S.M., Aly S.M. (2017). Fluorescent gold nanoclusters as pH sensors for the pH 5 to 9 range and for imaging of blood cell pH values. Microchim. Acta.

[28] Matus M.F., Häkkinen H. (2021). Atomically precise gold nanoclusters: Towards an optimal biocompatible system from a theoretical–experimental strategy. Small.

[29] Chang T.K., Cheng T.M., Chu H.L., Tan S.H., Kuo J.C., Hsu P.H., Su C.Y., Chen H.M., Lee C.M., Kuo T.R. (2019). Metabolic mechanism investigation of antibacterial active cysteine-conjugated gold nanoclusters in Escherichia coli. ACS Sustain. Chem. Eng..

[30] Ali R., Alfeneekh B., Chigurupati S., Saleh S.M. (2022). Green synthesis of pregabalin–stabilized gold nanoclusters and their applications in sensing and drug release. Arch. Pharm..

[31] Halawa M.I., Lai J., Xu G. (2018). Gold nanoclusters: Synthetic strategies and recent advances in fluorescent sensing. Mater. Today Nano.

[32] Goswami N., Luo Z., Yuan X., Leong D.T., Xie J. (2017). Engineering gold-based radiosensitizers for cancer radiotherapy. Mater. Horiz..

[33] Chen Y., Sun L., Liao F., Dang Q., Shao M. (2019). Fluorescent-stable and water-soluble two-component-modified silicon quantum dots and their application for bioimaging. J. Lumin..

[34] Borse S., Murthy Z.V.P., Park T.J., Kailasa S.K. (2021). Pepsin mediated synthesis of blue fluorescent copper nanoclusters for sensing of flutamide and chloramphenicol drugs. Microchem. J..

[35] Yang L., Hou P., Wei J., Li B., Gao A., Yuan Z. (2024). Recent Advances in Gold Nanocluster-Based Biosensing and Therapy: A Review. Molecules.

[36] Bai Y., Shu T., Su L., Zhang X. (2020). Fluorescent gold nanoclusters for biosensor and bioimaging application. Crystals.

[37] Kim H.M., Kim W.J., Kim K.O., Park J.H., Lee S.K. (2021). Performance improvement of a glucose sensor based on fiber optic localized surface plasmon resonance and anti-aggregation of the non-enzymatic receptor. J. Alloys Compd..

[38] Ding Y., Wang S., Li J., Chen L. (2016). Nanomaterial-based optical sensors for mercury ions. TrAC Trends Anal. Chem..

[39] Bigdeli A., Ghasemi F., Golmohammadi H., Abbasi-Moayed S., Nejad M.A.F., Fahimi-Kashani N., Jafarinejad S., Shahrajabian M., Hormozi-Nezhad M.R. (2017). Nanoparticle-based optical sensor arrays. Nanoscale.

[40] Yan X., Li H., Su X. (2018). Review of optical sensors for pesticides. TrAC Trends Anal. Chem..

[41] Grzelczak M., Pérez-Juste J., Mulvaney P., Liz-Marzán L.M. (2020). Shape control in gold nanoparticle synthesis. Colloidal Synthesis of Plasmonic Nanometals.

[42] Abdullah A., Altaf M., Khan H.I., Khan G.A., Khan W., Ali A., Bhatti A.S., Khan S.U., Ahmed W. (2018). Facile room temperature synthesis of multifunctional CTAB coated gold nanoparticles. Chem. Phys..

[43] Xie J., Zheng Y., Ying J.Y. (2009). Protein-directed synthesis of highly fluorescent gold nanoclusters. J. Am. Chem. Soc..

[44] Ma Y., Niu H., Zhang X., Cai Y. (2011). One-step synthesis of silver/dopamine nanoparticles and visual detection of melamine in raw milk. Analyst.

[45] Hu Y., Wang L., Song A., Hao J. (2018). Effect of Cationic Surfactants with Different Counterions on the Growth of Au Nanoclusters. Langmuir.

[46] Bakshi M.S. (2016). How surfactants control crystal growth of nanomaterials. Cryst. Growth Des..

[47] Dai C., Yang C., Yan X. (2018). Self-quenched gold nanoclusters for turn-on fluorescence imaging of intracellular glutathione. Nano Res..

[48] Murthy A.K., Stover R.J., Borwankar A.U., Nie G.D., Gourisankar S., Truskett T.M., Sokolov K.V., Johnston K.P. (2013). Equilibrium gold nanoclusters quenched with biodegradable polymers. ACS Nano.

[49] Zhang A., Guo W., Qi Y., Wang J., Ma X., Yu D. (2016). Synergistic effects of gold nanocages in hyperthermia and radiotherapy treatment. Nanoscale Res Lett..

[50] Rasheed T., Shafi S., Sher F. (2022). Smart nano-architectures as potential sensing tools for detecting heavy metal ions in aqueous matrices. Trends Environ. Anal. Chem..

[51] Zhang Y., Mollick S., Tricarico M., Ye J., Sherman D.A., Tan J.C. (2022). Turn-on fluorescence chemical sensing through transformation of self-trapped exciton states at room temperature. ACS Sens..

[52] Ali R., Alminderej F.M., Messaoudi S., Saleh S.M. (2021). Ratiometric ultrasensitive optical chemisensor film based antibiotic drug for Al (III) and Cu (II) detection. Talanta.

[53] Senila M., Cadar O., Miu I. (2020). Development and validation of a spectrometric method for Cd and Pb determination in zeolites and safety evaluation. Molecules.

[54] Klymus K.E., Merkes C.M., Allison M.J., Goldberg C.S., Helbing C.C., Hunter M.E., Jackson C.A., Lance R.F., Mangan A.M., Monroe E.M. (2020). Reporting the limits of detection and quantification for environmental DNA assays. Environ. DNA.

[55] Lin Y.C., Wu T., Lin Y.W. (2018). Fluorescence sensing of mercury (ii) and melamine in aqueous solutions through microwave-assisted synthesis of egg-white-protected gold nanoclusters. Anal. Methods.

